# Chemokine receptor CXCR7 antagonism ameliorates cardiac and renal fibrosis induced by mineralocorticoid excess

**DOI:** 10.1038/s41598-024-75789-0

**Published:** 2024-11-06

**Authors:** Bing H. Wang, Remy Robert, Francine Z. Marques, Niwanthi Rajapakse, Helen Kiriazis, Charles R. Mackay, David M. Kaye

**Affiliations:** 1 Heart Failure Research Group, Baker Heart and Diabetes Institute, St Kilda Rd Central, PO Box 6492, Melbourne, VIC 8008 Australia; 2 Biomarker Discovery, Baker Heart and Diabetes Institute, Melbourne, VIC Australia; 3 Department of Physiology, Biodiscovery Research Institute, Faculty of Medicine, Nursing and Health Services, Monash University, Clayton, VIC Australia; 4 Hypertension Research Laboratory, School of Biological Sciences, Monash University, Clayton, VIC Australia; 5 School of Biomedical Sciences, University of Queensland, Brisbane, QLD Australia; 6 Preclinical Cardiology, Microsurgery, and Imaging Platform, Baker Heart and Diabetes Institute, Melbourne, VIC Australia; 7 School of Pharmaceutical Sciences, Shandong Analysis and Test Center, Qilu University of Technology (Shandong Academy of Sciences), Jinan, 250014 China; 8 Monash-Alfred-Baker Centre for Cardiovascular Research, Faculty of Medicine, Monash University, Melbourne, VIC Australia

**Keywords:** Fibrosis, Mineralocorticoid excess, Chemokine receptors, CXCR7 antagonist, Drug discovery, Physiology, Cardiology, Nephrology, Pathogenesis

## Abstract

Cardiorenal fibrosis is a common feature of chronic cardiovascular disease and recent data suggests that cytokines and chemokines may also drive fibrosis. Here we tested the hypothesis that CXCR7, a highly conserved chemokine receptor, contributes to cardiac and renal fibrosis. We generated an anti-mouse CXCR7-specific monoclonal antibody (CXCR7 mAb) and tested its anti-fibrotic actions in cardiorenal fibrosis induced using the deoxycorticosterone acetate/uni-nephrectomy (DOCA-UNX) model. CXCR7 mAb treatment (10 mg/kg, twice weekly for 6 weeks) significantly attenuated the development of cardiac and renal fibrosis, and reduced fibrotic and inflammatory gene expression levels, in the absence of an effect on blood pressure. Immunohistochemical analysis demonstrated an increase in the vascular expression of CXCR7 in DOCA-UNX-treated mice. This study demonstrated that a CXCR7 mediated pathway plays a significant role in cardiac and renal fibrosis induced by DOCA-UNX treatment. Accordingly, antagonism of CXCR7 may provide a therapeutic opportunity to mitigate against fibrosis in the setting of mineralocorticoid excess.

**Supplementary Information:**

The online version contains supplementary material available at 10.1038/s41598-024-75789-0.

## Introduction

Fibrosis is a major contributor to progressive organ dysfunction and eventual failure including heart and kidney failure and causes significant mortality. In the heart, fibrosis results disruption of the coordinated myocardial excitation-contraction coupling in both systole and diastole and is a key feature of heart failure (HF) with reduced ejection fraction (HFrEF) and HF with preserved ejection fraction (HFpEF)^1^ and the extent and progression of fibrosis is associated with outcome^2^. Similarly, renal fibrosis is the hallmark of virtually all progressive kidney diseases and strongly correlates with the deterioration of kidney function leading to end-stage renal disease^3,4^. The renin angiotensin system has been clearly implicated in the pathogenesis of cardiorenal fibrosis, and experimental studies using mineralocorticoid antagonists^5–7^ and amiloride^8^ have demonstrated preventive capability. In clinical practice however, evidence for an antifibrotic action of renin-angiotensin-aldosterone inhibition is more controversial^9,10^.

In addition to their roles in leukocyte trafficking, chemokines are crucial participants involved in the regulation of organ fibrosis through immune cell recruitment and activation of their receptors^11^. As such they may also be targets for intervention as potential anti-fibrotic therapies. Chemokines are divided into homeostatic and inflammatory groups based on their expression patterns and the types of cells they attract. Some members of the chemokine family, including chemokine CXC ligand 12 (CXCL12 / SDF-1), have both homeostatic and inflammatory roles by both constitutive expression and induction in inflamed tissue^12^. Induction of several inflammatory chemokines is associated with fibrosis^12^. The proinflammatory action of chemokines plays important roles in the pathology of tissue fibrosis in the heart, kidney, and other organs^13–15^. Chemokines attract inflammatory cells in the blood to infiltrate the heart and kidney after injury and the release of inflammatory cytokines causes the proliferation of fibroblasts and further differentiation to myofibroblasts and synthesis of ECM leading to fibrosis^12^.

A growing body of evidence suggests that CXCL12 may be implicated in the pathogenesis of fibrosis in several different organs^16^. CXCL12 exerts a wide range of actions on all cell types involved in cardiac remodeling including inflammatory leukocyte recruitment, angiogenesis (through recruitment of progenitors), and cardiomyocyte survival^16^. CXCL12 interacts with its cognate receptor, CXCR4, and is essential for the development of the heart, brain, and blood vessels^17–22^. Its fibrogenic actions may involve direct effects on fibroblast migration or activation of fibrogenic macrophages, and our recent studies in experimental hypertension and HF models confirm the pathogenic role of CXCR4 in cardiac and renal fibrosis^23–25^.

CXCL12 has also been shown to bind to CXCR7 and may exert actions independent of CXCR4^26^, although observations from several studies have not been consistent^26–28^. CXCR7 is known to be expressed in both the heart^29^ and kidney^30^. In addition to binding CXC12, with consequent activation of ERK-mediated signalling, CXCR7 may also potentially function as a scavenger receptor of CXCL12 that controls its local levels^31^. Given uncertainty about the role of CXCR7 in cardiorenal pathology, we therefore aimed to investigate the anti-fibrotic effects of CXCR7 inhibition using the deoxy-corticosterone acetate - nephrectomy- saline (DOCA-UNX) model of cardiorenal fibrosis^25^.

## Materials and methods

All experimental protocols were approved by the Alfred Medical Research and Education Precinct (AMREP) Animal Experimentation Ethics Committee under the guidelines of the National Medical and Health Research Council of Australia (Ethics approval number: E/1567/2015/B). All methods and procedures were carried out in accordance with relevant guidelines and regulations. The study design conforms with the ARRIVE guidelines.

### Generation of anti-CXCR7 monoclonal antibodies

mAbs reactive with CXCR7 were generated by immunizing *cxcr7* KO mice with 10^7^ L1.2 human CXCR7 transfected cells, intraperitoneally (i.p), five to six times at 2-week intervals. The final immunization was injected intravenously (i.v). 4 days later, the spleen was removed, and cells were fused with the SP2/0 cell line as described^32^. To assess the reactivity and specificity of mAbs against transfected cells, we used indirect immunofluorescence staining and flow cytometry. Cells were washed once with PBS and resuspended in 100 µl PBS containing 2% BSA and 0.1% sodium azide (staining buffer) and purified antibody. After 30 min at 4 °C, cells were washed twice with staining buffer and resuspended in 50 µl PE-conjugated anti-human IgG (Jackson ImmunoResearch Laboratories) diluted 1:500 in staining buffer for the detection of humanized mAbs or 50 µl PE-conjugated anti-mouse IgG (Jackson ImmunoResearch Laboratories) for the detection of mouse mAbs. After incubating for 20 min at 4 °C, cells were washed twice with staining buffer and analyzed on an LSRII flow cytometer (BD Biosciences). 7-AAD staining was used to exclude dead cells^32^.

### Confirmation of inhibitory action of CXCR7 mAb

The PathHunter™ protein complementation assay (DiscoveRx Corporation) was performed according to the manufacturer’s instructions to confirm the antagonistic activity of the anti-CXCR7 antibody. The cells were plated in 384-well plates at 10,000 cells per well and cultured for 24 h at 37 °C, 5% CO2. Then, the cells were incubated at 37 °C for 90 min with anti-hCXCR7 antibodies and CXCL12 (Peprotech) ligand at EC90 concentration (2 nM) in assay media. The substrate was then added and incubated at RT for 60 min before measuring luminescence using a plate reader (Perkin Elmer). Samples were assayed in triplicate.

### Ligand binding assay

Human CXCL 12 (SDF-1 a) (“ligand”) was obtained from Peprotech (New Jersey, USA). ^125^I- CXCL12 was purchased from Perkin­Elmer (Boston, MA, USA), with a specific activity of 2200 Ci/mM. Cells were washed once in binding buffer (50 mM Hepes, pH 7.5, 1 mM CaCI, 5 mM MgCl2, 0.5% BSA) and resuspended in binding buffer at a concentration of 2.5 × 10^6^ cells/ml. Cold Purified monoclonal (cold competitor) was added to a 96-well plate followed by an equal volume (40 µI) binding buffer containing 1 × 10^5^ cells. Cells and competitors were preincubated at room temperature for 15 min. Then radiolabelled ligand (final concentration 2 nM) was added to each well to give a final reaction volume of 120 µI. After a 60-min incubation at room temperature, the cells were washed three times with 1 ml of binding buffer containing 150 mM NaCl. The radioactivity in the cell pellets was counted in a TopCount liquid scintillation counter (Packard). Non-specific background binding was calculated by incubating cells without a radiolabelled ligand. Samples were assayed in triplicate.

### Animal model and in vivo study design

The deoxycorticosterone - uni-nephrectomy model of cardiorenal fibrosis was established in male C57BL/6 mice (6 weeks old, obtained from the Precinct Animal Centre, Baker Heart and Diabetes Institute) as previously reported^25^. Briefly, under isoflurane (inhalation; 2% induction and 1-1.5% maintenance; throughout surgery) anaesthesia, a left lateral abdominal incision was performed to access the left kidney, which was removed following ligation of the renal vessels and ureter. Mice were also implanted with a 21-day slow-release pellet in the right flank containing either deoxy-corticosterone acetate (DOCA), Innovative Research of America, Sarasota, FL, USA) or a placebo containing the inert carrier while anaesthetised with isoflurane (*n* = 5 ~ 7 per group). After 21 days, an additional DOCA) or placebo pellet was implanted in the left flank for the remainder of the experiment (a total of 6 weeks of DOCA treatment), when mice were euthanized at 12 weeks of age. Controls did not undergo uninephrectomy. All animals received isotonic saline (10 g NaCl/L) for 6 weeks from starting the DOCA-UNX treatment. The animals were monitored, and their body weights were measured weekly for a period of 6 weeks.

Mice were randomly allocated to receive either purified CXCR7 antagonist monoclonal antibody (anti-CXCR7 mAb, clone 10D1, 10 mg/kg) or control with intraperitoneal (IP) injection twice per week for 6 weeks, started at the first day of the protocol. The treatment groups are as follows: **Control**: Sham-operated, placebo pellet, IP injection with Isotype control clone is C1.18.4 from BioXcell (*n* = 7); **Control + CXCR7** antagonist: placebo pellet, IP injection with anti-CXCR7 mAb (*n* = 7); **DOCA-UNX**: DOCA-UNX pellet, IP injection with saline (*n* = 7); and **DOCA-UNX + CXCR7** antagonist: DOCA-UNX pellet, IP injection with anti-CXCR7-blocking mAb for 6 weeks (*n* = 6).

### Cardiac functional measurements

At the end of the study, M-mode echocardiographic imaging of the heart was performed under isoflurane anaesthesia with a 15-MHz linear transducer as previously reported^25^. Off-line image analyses were performed in a blinded fashion. On the following day, arterial blood pressures and left ventricle (LV) pressures were determined using a 1.4-F Millar Mikro-Tip^®^ transducer catheter (Millar Instrument Co.) under anaesthesia as described above.

### Kidney function measurement

Mice were placed in metabolic cages at the end of the experiment to collect 24-h urine samples for creatinine clearance. Creatinine concentration in plasma and urine was assessed as previously described^33^. Creatinine clearance was estimated using the following formula: (urine creatinine concentration x urine flow rate)/plasma creatinine concentration.

### Morphometry and histological analysis

Animals were humanly killed at the end of the experiments by anaesthesia (2% isoflurane inhalation) and the tissues of the heart, lung, and kidney were harvested. The heart, kidney, and lung tissues were weighed, and tissue sections were either fixed in 10% formalin in PBS for histological or snap-frozen in liquid nitrogen for molecular biological analyses. Four-micron paraffin sections were stained with Masson’s Trichrome. The extent of perivascular and interstitial fibrosis was quantified in ten randomly selected fields per animal from images captured using an Olympus BH2 microscope and analysed with Image Pro Plus software (Adept Electronic Solutions Pty Ltd, Moorabbin, Australia). The results were expressed as a percentage of the blue area on each screen at a magnification of 400x. The perivascular and interstitial collagen volume fractions of the Masson’s Trichrome stained tissue were measured separately. All collagen surrounding an intramyocardial coronary artery was considered perivascular collagen. The investigator responsible for the morphometric analysis was blinded to each experimental group.

### Tissue expression of CXCR7: immunohistochemistry staining

Five-micron thick cryostat sections of the heart were air-dried and fixed with 4% paraformaldehyde in PBS solution. Endogenous peroxidases were then quenched with 3% H_2_O_2_ for 15 minutes and incubated with 10% goat serum for 60 minutes to block non-specific binding. Sections were incubated with rabbit polyclonal antibody to CXCR7 (1:500; ab72100, Abcam, Cambridge, UK) overnight in a humidified chamber at 4°C. On the following day, sections were rinsed and incubated with anti-rabbit horseradish peroxidase-conjugated secondary antibody (1:500; ab6721, Abcam, Cambridge, UK). Sections were then incubated with 3,3’-diaminobenzidine tetrahydrochloride (D4293-50SET; Sigma, St. Louis, Missouri, USA) for 5 min. Slides were then dehydrated and mounted with DPX.

### Tissue expression of CXCR7: immunofluorescence staining

Five-micron thick cryostat sections of the heart were air-dried, fixed in cold acetone, and then incubated with CAS-Block (Invitrogen, Frederick, MD, USA) for reducing nonspecific background staining. Sections were incubated, as indicated, with the following antibodies: rabbit polyclonal anti-CXCR7 (1:500; ab72100, Abcam, Cambridge, UK); mice monoclonal anti α-smooth muscle actin (1:100; Sigma, Saint Louis, USA); or an endothelial cell antibody (rat, anti-mouse CD31,1:100; BD Biosciences, Franklin Lakes, NJ, USA) overnight in a humidified chamber at 4 °C. On the following day, sections were rinsed and incubated with goat anti-rabbit IgG (H + L) Alex Fluor 488 and goat anti-mouse IgG (H + L) Alex Fluor 647 secondary antibody (1:250; A32731&A32728, I*nvitrogen*, Paisley, UK). Slides were then mounted in VECTASHIELD antifade mounting medium (Vector Laboratories, Burlingame, CA, USA) and viewed with a Zeiss Axiovert 200 M microscope.

### Fibrotic and inflammatory gene expression quantification

Real-time PCR (qPCR) was performed to determine collagen type I (*Cola1a1*) and III (*Col3a1)*, and *TGFβ* mRNA expression in mouse heart and kidney tissues. Total RNA was isolated by TRIzol (Thermo Fisher Scientific) and reverse transcribed to cDNA using the High-Capacity cDNA Reverse Transcription Kit (Thermo Fisher Scientific). Amplification reactions used the SYBR Green Fast PCR master mix in a QuantStudio qPCR instrument (both from Thermo Fisher Scientific) using a 20ng cDNA template. Samples were run in duplicate and the primers sequences for qPCR are detailed in Supplementary Table S1. Expression was analysed relative to the housekeeping gene *Gapdh*. All primers were designed on exon-exon junctions. Gene expression levels were determined by calculating the ∆∆Ct value for each reaction and data was expressed as a fold difference compared to the control.

### Expression of CXCR7 in human aortic smooth muscle and endothelial cells, and ventricular fibroblasts

Normal human smooth muscle and endothelial cells, and ventricular fibroblasts were purchased from ATCC and cultured according to ATTC’s recommendation. Cells were seeded in chamber slides and incubated overnight. Cell culture media were changed to normal serum-containing media for further 24 h before being fixed with formalin. Cells were stained with CXCR7 or αSMA antibodies with protocols previously described^17^ and viewed with a Zeiss Axiovert 200 M microscope.

### Statistical analyses

All data were presented as the mean ± SEM and the statistical analyses were carried out using the Statistical Package for the Social Sciences (SPSS) software (SPSS 22 for Windows, SPSS, Chicago, USA). Between-group comparisons were performed with GraphPad Prism (version 9) using two-way ANOVA with correction for multiple comparisons using a false discovery rate (FDR). A p-value of < 0.05 was considered statistically significant.

## Results

### Generation and characterization of a novel anti-CXCR7 mAb

**Fig. 1:**
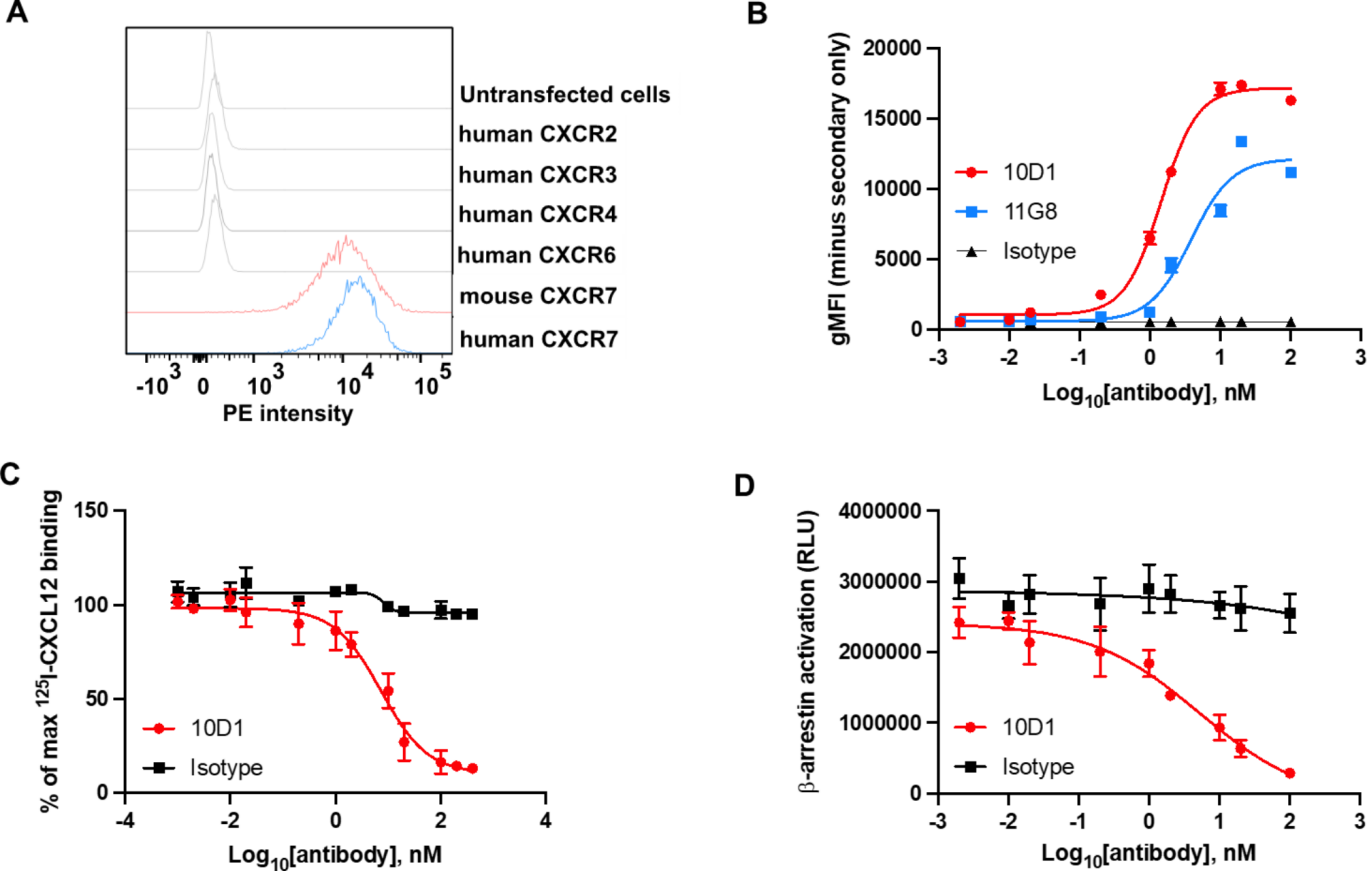
Characterization of anti-CXCR7 mAb. (**A**) 10D1 staining of various L1.2 transfectants. Stable L1.2 transfectants expressing either hCXCR2, hCXCR3, hCXCR4, hCXCR6, hCXCR7, mCXCR7 were stained with the anti-CXCR7 mAb 10D1. (**B**) The reactivity of 10D1 and 11G8 was assessed by flow cytometry using increasing concentrations of purified mAbs. (**C**) Inhibition of binding of CXCR3-positive cells to radiolabelled CXCL12 by anti-CXCR7 antibody. hCXCR7-transfected cells were incubated with ^125^I-labeled human CXCL12 in the presence of increasing concentrations of 10D1 antibody. (**D**) Antagonistic activity of 10D1 in β-arrestin assays.

CXCR7 mAbs were produced by immunizing KO mice with the murine pre–B cell lymphoma line, L1.2, which expresses high levels of transfected human CXCR7. One mAb, termed 10D1 reacted both with hCXCR7 and mCXCR7-transfected L1.2 cells, but not with L1.2 cells expressing other chemokine receptors (Fig. 1A). 10D1 shows a better reactivity than the commercially available antibody 11G8 (Fig. 1B), as well as vastly superior blocking ability (below). To evaluate the ability of our mAb to antagonize CXCR7, ligand binding, and β-arrestin recruitment assays were performed (Fig. 1C and D). In both assays, 10D1 was able to significantly inhibit CXCL12 ligand binding and signalling which was used in this study.

### Characteristics of the animal model

As shown in Table 1, DOCA-UNX administration significantly increased heart and kidney weights (HW and KW), by 29% and 90%, respectively (both *p* < 0.05 vs. control), together with their ratios to body weight (BW, *p* < 0.05 vs. control, Table 1). Lung weight (LW) and LW/BW ratios in the DOCA-UNX-treated animals tended to be higher than in the control animals but the difference was not significant. CXCR7 mAb treatment (DOCA-UNX + CXCR7) reduced heart, kidney, and lung weights, and their ratios to BW compared to DOCA-UNX-treated mice, although the differences were not statistically significant (Table 1). CXCR7 mAb treatment in control mice (Control + CXCR7) had no significant effects on body weight, heart weight, lung weight, or kidney weight.

**Table 1:** Characteristics of the animals and effects of CXCR7 mAb treatment.

	Control	Control + CXCR7	DOCA-UNX	DOCA-UNX + CXCR7
Group size	7	7	6	6
Body weight (BW) (g)	27.9 ± 0.8	28.8 ± 0.6	26.5 ± 0.9	26.3 ± 0.9
Heart weight (HW) (mg)	144 ± 8	153 ± 9	186 ± 14*	175 ± 7
HW/BW (mg/g)	5.2 ± 0.2	5.3 ± 0.2	7.07 ± 0.7*	6.7 ± 0.4
Kidney weight (KW) (mg)	306 ± 24	310 ± 15	582 ± 35*	489 ± 30*
KW/BW (mg/g)	10.9 ± 0.6	10.7 ± 0.4	21.9 ± 1.0*	16.3 ± 3.0*
Lung weight (LW) (mg)	168 ± 8	188 ± 7	198 ± 22	177 ± 7
LW/BW (mg/g)	6.0 ± 0.2	6.5 ± 0.2	7.6 ± 1.0	6.7 ± 0.2
Tibia length (TL) (mm)	17.4 ± 0.1	17.4 ± 0.1	16.9 ± 0.1*	17.2 ± 0.2
Plasma Cr (µg/dL)	61 ± 5	60 ± 8	43 ± 6*	45 ± 6*
Urine volume (ml) (24 h)	1.4 ± 0.3	1.3 ± 0.3	28.0 ± 4.7*	6.2 ± 1.8*^#^

All data were presented as the mean ± SEM. *p < 0.05 vs. Control. ^#^*p* < 0.05 vs. DOCA-UNX.

### Effects of CXCR7 mAb treatment on cardiac structure and function

**Fig. 2:**
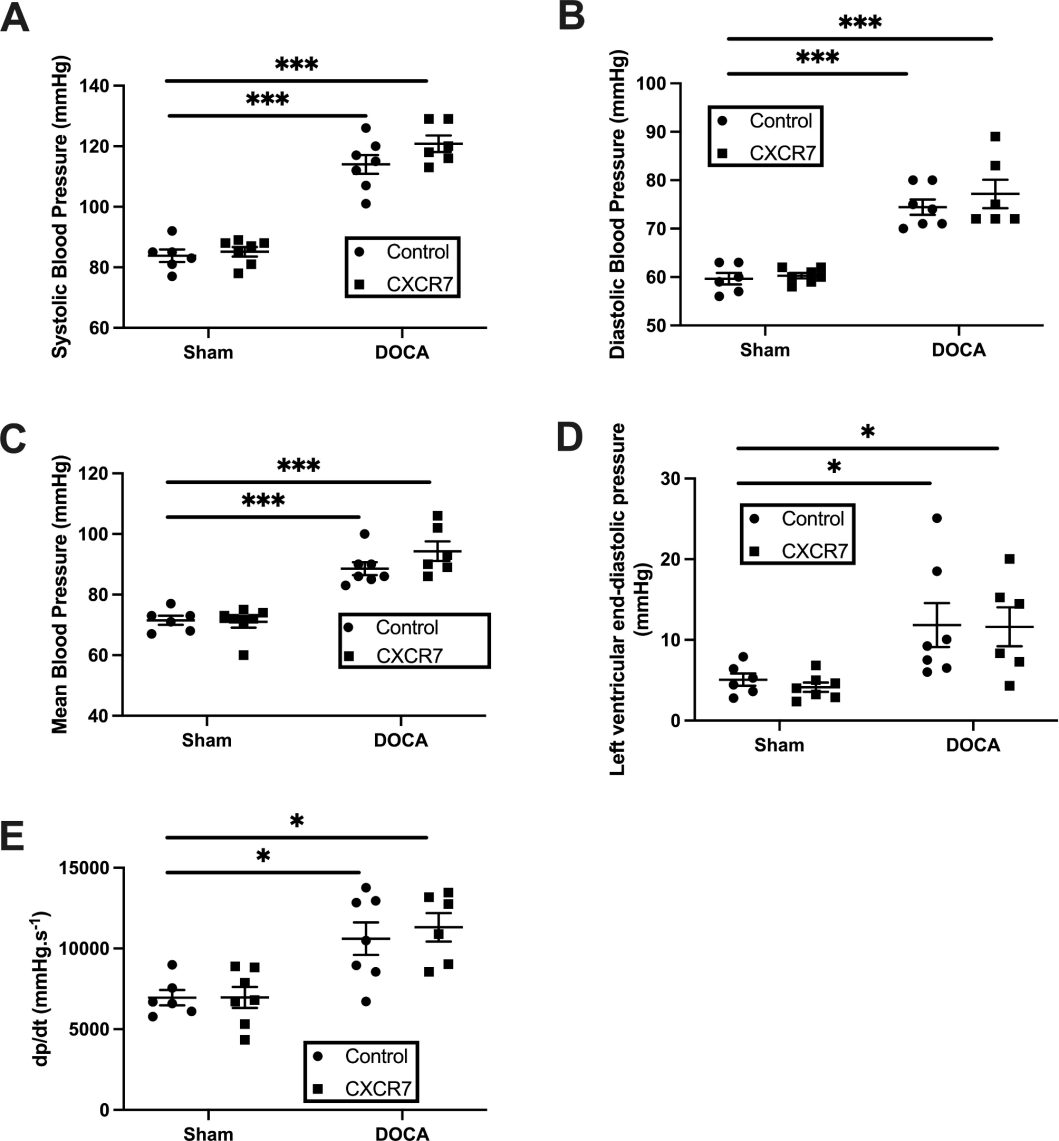
Haemodynamic measurements: Blood pressure (systolic, diastolic, mean pressure) (**A** to **C**) and left ventricular end-diastolic pressure (**D**,**E**) measured by cardiac catheterization in control mice (Sham-operated with isotype injection control; *n* = 6) and mice treated with CXCR7 (*n* = 7), DOCA-UNX (*n* = 7), or CXCR7 plus DOCA-UNX (*n* = 6). Data are presented as the mean ± SEM. *p < 0.05, ***p < 0.001 vs. Control.

DOCA-UNX treatment was associated with a significant increase in the thickness of the interventricular septum at end-diastole (IVSd, *p* < 0.05 vs. control), and the LV posterior wall (LVPWd, *p* < 0.05, vs. Control) compared to control mice (Table 2). DOCA-UNX treatment did not affect LV fractional shortening. Treatment with CXCR7 mAb (DOCA-UNX + CXCR7) attenuated the increase in LV posterior wall thickness (LVPWd, *p* < 0.05) but had no effects on other LV measurements including LV chamber size or fractional shortening (Table 2). There was no demonstrable effects of treatment with CXCR7 mAb in control mice (Table 2). DOCA-UNX treatment was associated with a significant increase in blood pressure which was not altered by CXCR7 mAb treatment (Fig. 2A to C). CXCR7 mAb treatment also had no effect on blood pressure in control mice (Control + CXCR7) (Fig. 2A to C). Furthermore, CXCR7 mAb treatment had no effect on LV end-diastolic pressure (LVEDP) or LV dP/dt (Fig. 2D, E).

**Table 2:** Echocardiographic measurements and cardiac function.

	Control	Control + CXCR7	DOCA-UNX	DOCA-UNX + CXCR7
Group size	6	6	6	6
IVSd, mm	0.68 ± 0.02	0.66 ± 0.02	0.92 ± 0.05*	0.86 ± 0.05*
LVIDd, mm	4.07 ± 0.07	4.43 ± 0.18	4.36 ± 0.27	4.49 ± 0.16
LVIDs, mm	2.87 ± 0.10	3.11 ± 0.20	3.15 ± 0.26	3.22 ± 0.19
LVPWd, mm	0.72 ± 0.02	0.69 ± 0.03	0.83 ± 0.03*	0.71 ± 0.02^#^
FS (%)	30 ± 2	30 ± 2	28 ± 3	29 ± 2
Heart Rate	468 ± 20	460 ± 18	464 ± 21	455 ± 26

All data were presented as the mean ± SEM. A p-value of < 0.05 was considered significant. *p < 0.05 vs. Control (Sham-operated with isotype injection control). ^#^*p* < 0.05 vs. DOCA-UNX.

**Fig. 3:**
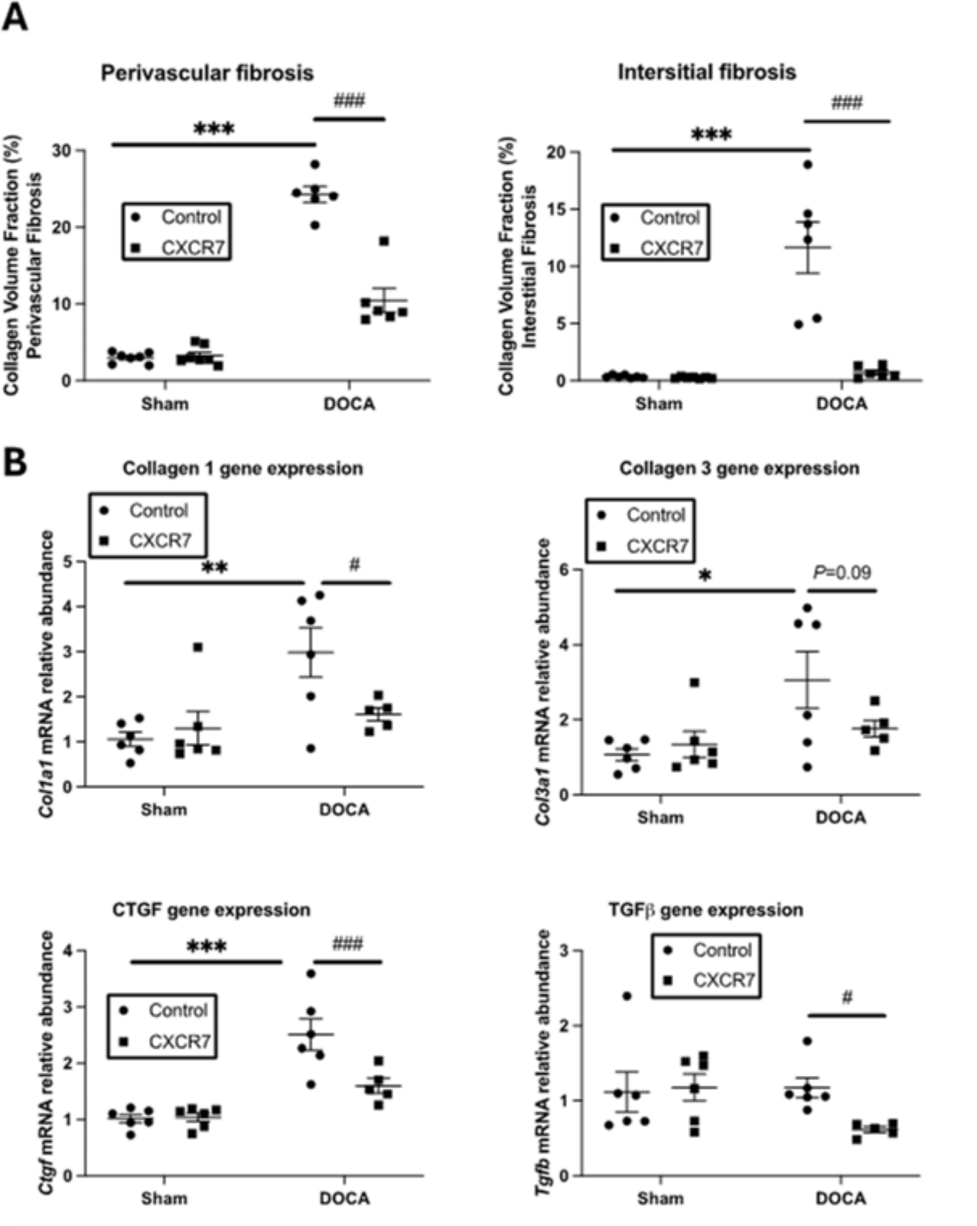
Cardiac fibrosis and fibrotic gene expression. (**A**) Graph representing perivascular and interstitial collagen volume fraction in Control (Sham-operated with isotype injection control) (*n* = 7), Control + CXCR7 (*n* = 7), DOCA-UNX (*n* = 6) and DOCA-UNX + CXCR7 (*n* = 6) mice. Data are presented as the mean ± SEM. ***P < 0.001 vs. Control. ^###^*P* < 0.001 vs. DOCA-UNX. (**B**) Fibrotic gene expression in the Control (*n* = 6), Control + CXCR7 (*n* = 6), DOCA-UNX (*n* = 6), and DOCA-UNX + CXCR7 mice (*n* = 5). Data are presented as the mean ± SEM. *P < 0.05, **p < 0.01, ***p < 0.001 vs. control. ^#^*P* < 0.05, ^###^*p* < 0.001 vs. DOCA-UNX.

DOCA-UNX treatment significantly elevated collagen deposition in the heart both in the perivascular and interstitial spaces in comparison to control (Fig. 3A and B). CXCR7 mAb treatment significantly reduced the perivascular and interstitial collagen deposition compared to DOCA-UNX-treated animals. DOCA-UNX treatment also significantly increased fibrotic gene expression levels, including *Col1a1*, *Col3a1*, and *Ctgf*, compared to control mice (Fig. 3B). Treatment with CXCR7 mAb significantly reduced *Col1a1 and Ctgf* gene expressions whilst *Col3a1* gene expression was reduced but did a not reach significance level (*p* = 0.09) as compared to DOCA-UNX-treated animals. CXCR7 mAb treatment also significantly reduced *Tgfβ* gene expression in the hearts of mice exposed to DOCA-UNX (Fig. 3C). CXCR7 mAb treatment had no effects on both collagen deposition and fibrotic gene expression in the hearts of control mice.

### Effects of CXCR7 mAb treatment on renal structure and function

**Fig. 4:**
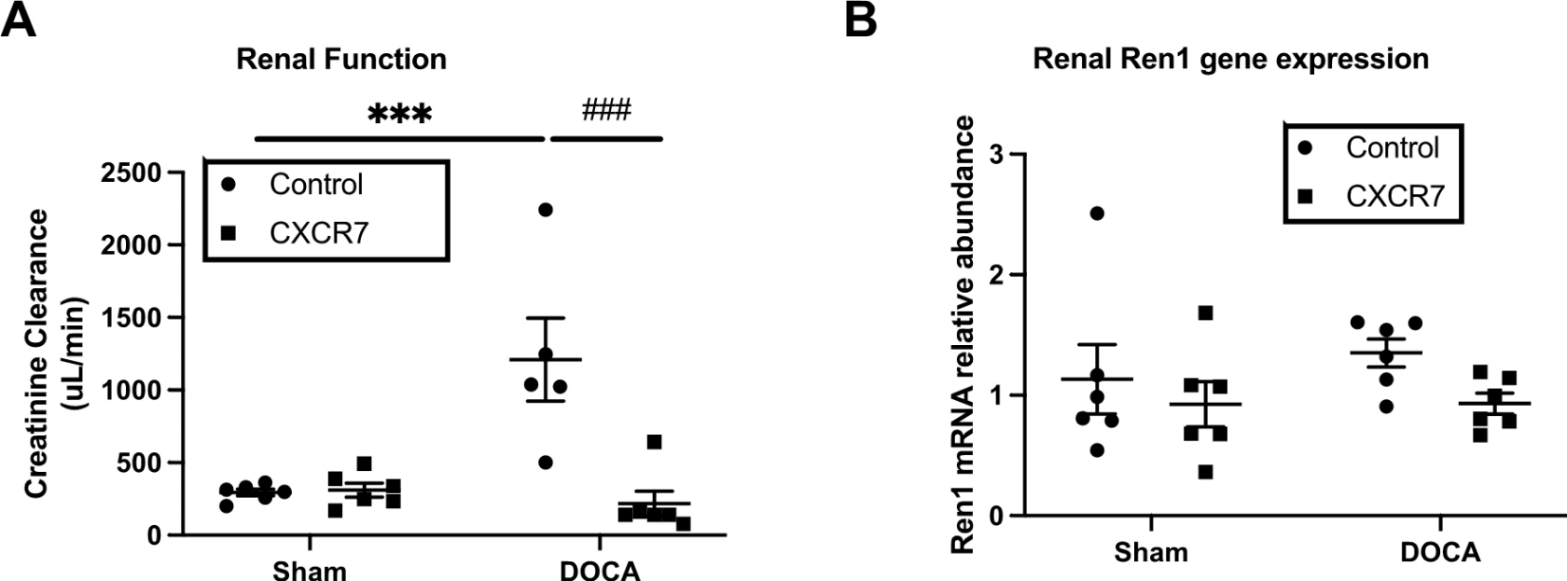
Kidney functional results. The graph presents kidney functional data as measured by GFR (**A**) and *Ren1* gene expression (**B**) in the Control (Sham-operated with isotype injection control) (*n* = 6), Control + CXCR7 (*n* = 6), DOCA-UNX (*n* = 5 ~ 6) mice, and DOCA-UNX + CXCR7 (*n* = 6). Data are presented as mean ± SEM. ***p < 0.001 vs. control, ^###^*p* < 0.001 vs. DOCA-UNX.

Urine volumes were markedly increased in DOCA-UNX mice compared to the control animals and CXCR7 treatment significantly reduced urine volumes (*p* < 0.05 vs. DOCA-UNX), (Table 1). Plasma creatinine was reduced in the DOCA-UNX groups as has been previously reported^34^. Consistent with increasing urine output (Table 1), DOCA-UNX-treated animals had significantly higher GFR compared to control animals (Fig. 4A). CXCR7 mAb treatment significantly attenuated the hyperfiltration profile towards levels equivalent to the control animals. CXCR7 mAb had no effect on GFR in control animals (Fig. 4A). Both DOCA-UNX treatments with or without CXCR7 treatment did not significantly alter the *Ren1* gene expression level (Fig. 4B).

**Fig. 5:**
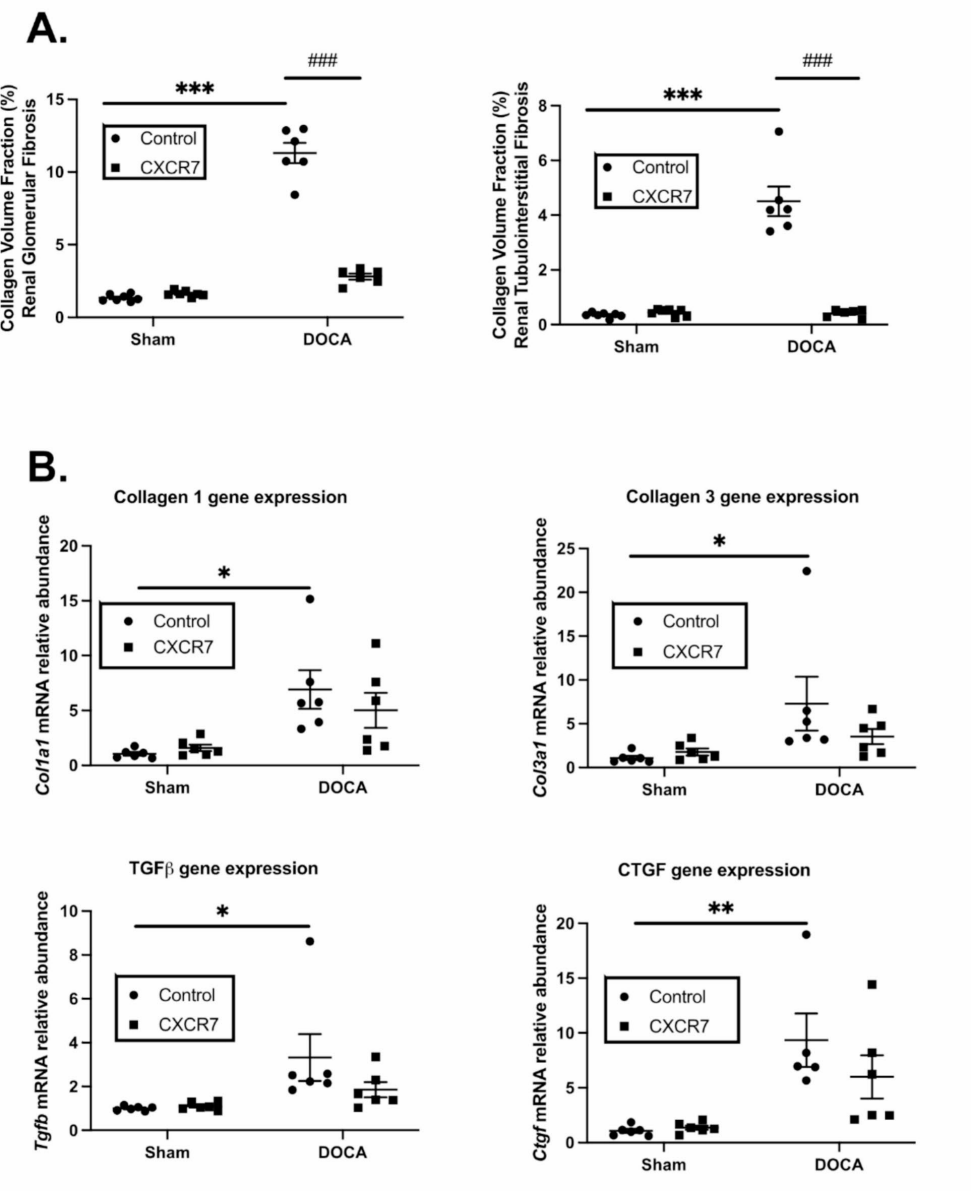
Collagen deposition and fibrotic gene expression in the kidney. (**A**) Graph represents glomerular and tubulointerstitial collagen volume fraction in the Control (*n* = 7), Control + CXCR7 (*n* = 7), DOCA-UNX (*n* = 6), or DOCA-UNX + CXCR7 (*n* = 6) mice. Data are presented as mean ± SEM ***p < 0.001 vs. Control. ^###^*p* < 0.001 vs. DOCA-UNX. (**B**) Kidney fibrotic gene expression in the Control (Sham-operated with isotype injection control) (*n* = 6), Control + CXCR7 (*n* = 6), DOCA-UNX (*n* = 5–6)mice, and DOCA-UNX + CXCR7 (*n* = 6) mice. Data are presented as mean ± SEM *P < 0.05, **P < 0.01 vs. Control.

Mineralocorticoid excess with DOCA-UNX treatment significantly elevated collagen deposition (fibrosis) of the kidney both in the glomerular and tubulointerstitial spaces in comparison to control animals (Fig. 5A). CXCR7 mAb treatment significantly reduced the collagen deposition in the kidney of the animals as compared to DOCA-UNX-treated mice. DOCA-UNX treatment significantly elevated fibrotic *Col1a1*, *Col3a1*,* Tgfb*, and *Ctgf* gene expression levels in the kidney as compared to the control animals (Fig. 5B). CXCR7 mAb treatment had no significant effect on these fibrotic gene expression levels as compared to DOCA-UNX-treated mice. CXCR7 mAb treatment had no detectable effects on collagen deposition and fibrotic gene expression levels in the kidneys of control mice (Fig. 5B).

### Effects of CXCR7 mAb treatment on inflammatory gene expression

**Fig. 6:**
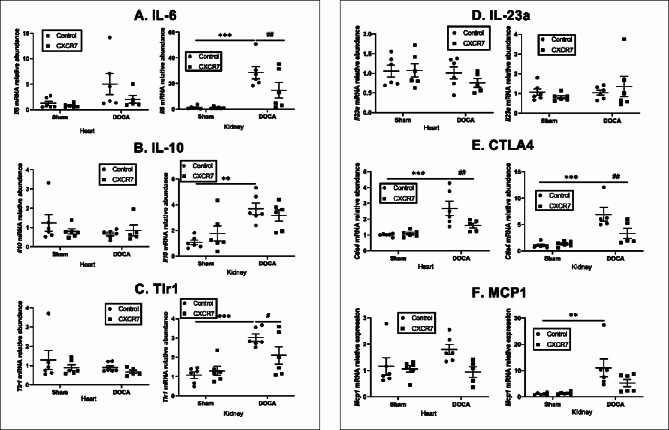
Heart and kidney inflammatory gene expression in the Control (*n* = 6), Control + CXCR7 (*n* = 6), DOCA-UNX (*n* = 5–6) or DOCA-UNX + CXCR7 (*n* = 5–6) mice. Data are presented as mean ± SEM. **p < 0.01, ***p < 0.001 vs. Control. ^#^*p* < 0.05, ^##^*p* < 0.01 vs. DOCA-UNX.

Consistent with the inflammatory aspect of mineralocorticoid induced hypertension^35^, we found that *IL6* gene expression was significantly increased in the kidney of DOCA-UNX-treated mice. CXCR7 mAb treatment significantly reduced *IL6* gene expression levels in the kidney only (Fig. 6A). Interestingly, DOCA-UNX treatment also elevated *IL10* gene expression levels compared to control mice and CXCR7 treatment had no effect on *IL10* gene expression (Fig. 6B). *Tlr1* mRNA was significantly higher in the kidney, but not in the heart, of DOCA-UNX-treated mice, and CXCR7 mAb treatment significantly reduced it (Fig. 6C), but there were no changes in *Il23a* mRNA (Fig. 6D). *Ctla4* mRNA expression was significantly increased in the heart and kidney of DOCA-UNX-treated mice, and CXCR7 mAb treatment significantly attenuated it (Fig. 6E). DOCA-UNX treatment significantly increased MCP1 gene expression in the kidney, but the increase was not significant in the heart, and CXCR7 treatment reduced MCP1 gene expression in both the kidney and the heart (Fig. 6F).

### Effects of CXCR7 mAb treatment on CXCR7 expression

**Fig. 7:**
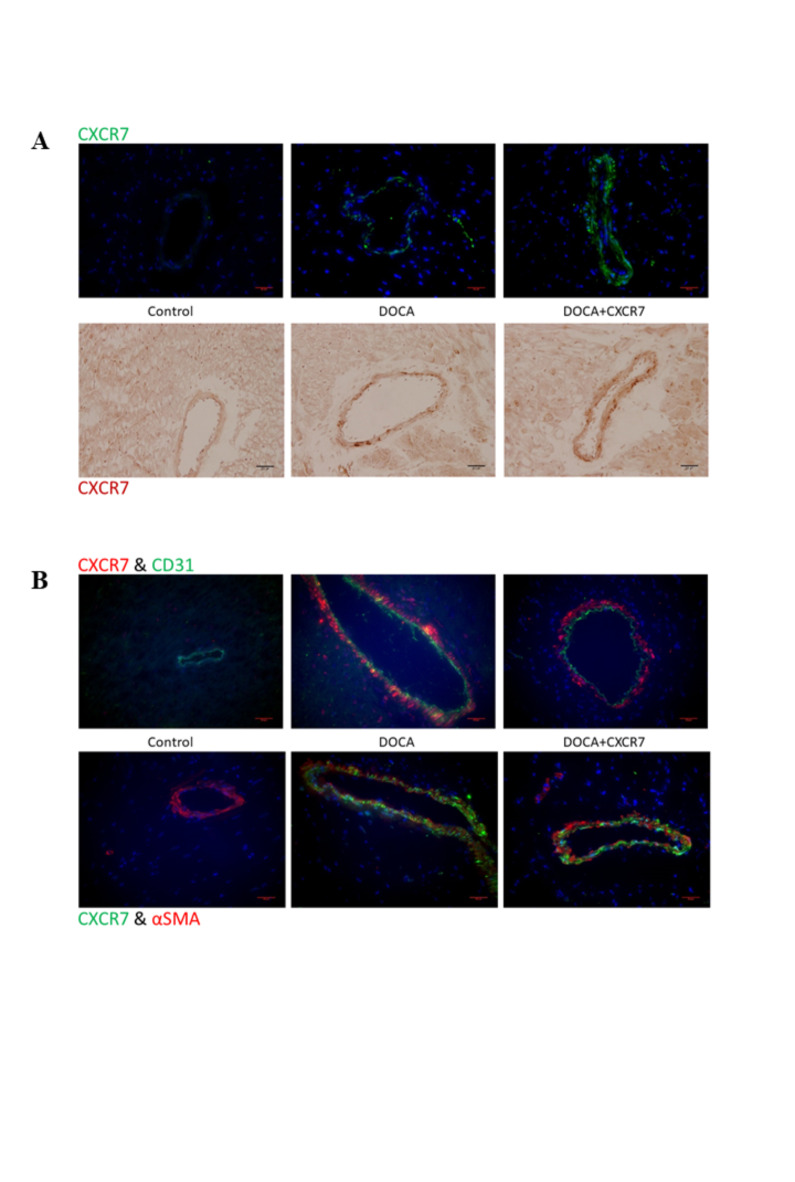
(**A**) Representative immunohistochemical and immunofluorescence staining of CXCR7 in the perivascular region of myocardial sections from Control, DOCA-UNX or DOCA-UNX + CXCR7 mice. (**B**) Representative double-immunofluorescence staining of CXCR7 with CD31 and αSMA in the perivascular region of Control (Sham-operated with isotype injection control), DOCA-UNX or DOCA-UNX + CXCR7 mice. Staining was performed as described in the Method sections and the representative images are at 400X magnification.

We next characterised the expression of CXCR7 in the context of DOCA-UNX induced fibrosis, by specifically addressing its distribution in the cardiac perivascular fibrosis paradigm using both immunohistochemistry and immunofluorescence staining. CXCR7 was readily detectable in DOCA-UNX and DOCA-UNX + CXCR7 treated animals when compared to control animals (Fig. 7A), with immunostaining suggesting a modest increase in perivascular CXCR7 expression in the presence of the CXCR7 mAb. Immunostaining of myocardial sections (Fig. 7B, Supplementary Fig. 5) suggested that the distribution of CXCR7 aligned with myofibroblasts rather than with endothelial cells.

## Discussion

CXCL12 and its receptors CXCR4 and CXCR7 have been demonstrated to exert a pleiotropic series of actions as mediators of development, chemotaxis, and disease pathogenesis. Recently, the potential therapeutic utility of manipulating the CXCL12-CXCR4/7 axis has become a therapeutic target of interest^36^. Utilising DOCA-UNX-induced mineralocorticoid excess induced hypertension and fibrosis in C57BL/6 mice, we have shown that CXCR7 antagonist monoclonal antibody treatment significantly attenuated cardiac and renal fibrosis, and related fibrotic gene expression without affecting blood pressure, cardiac chamber sizes and function. CXCR7 antagonism attenuated the increase in posterior wall thickness in the heart induced by DOCA-UNX. CXCR7 antagonism also significantly reversed the increase of GFR due to DOCA-UNX treatment.

CXCR7 antagonism did not affect blood pressure and the chamber sizes but significantly reduced LV posterior wall at end-diastole (LVPWd). In addition, this study also showed that CXCR7 mAb treatment significantly reduced both perivascular and interstitial fibrosis, and related fibrotic gene expression in the heart of DOCA-UNX-treated mice indicating DOCA-UNX-induced cardiac fibrosis at least in part *via* the CXCR7 pathway. Despite the attenuation of myocardial fibrosis, CXCR7 mAb treatment did not have a significant effect on cardiac function, potentially due to the relatively short experimental period.

This study showed that CXCR7 mAb treatment significantly attenuated renal hyperfiltration. There was a significant increase in kidney weight in DOCA-UNX mice,  and CXCR7 mAb treatment reduced kidney weight, but it did not reach a significant level. We did not measure the effect of CXCR7 mAb on urinary Na^+^. CXCR7 mAb treatment also significantly reduced renal fibrosis.

CXCR7 mAb treatment abolished inflammatory responses including reduction of inflammatory cytokine IL-6 gene expression and the expression levels of *CTLA4* and *MCP1* indicating attenuation of immune cell infiltration. A previous study has shown that *CTLA4* blockade with CTLA4-Ig abrogated the activation of circulating T cells, T-cell cytokine production, and vascular T-cell accumulation due to the hypertensive stimuli with DOCA-UNX or angiotensin II^37^. Unlike CTLA4-Ig, CXCR7 mAb treatment has no effect on blood pressure despite a significant reduction of *CTLA4* gene expression observed. Thus, CXCR7 mAb may exert its anti-inflammatory effects *via* the inactivation of immune cell responses independent of blood pressure.

Furthermore, histology staining showed that CXCR7 is expressed in vessels of the heart and kidney. In vitro staining showed CXCR7 expression in cardiac fibroblasts, smooth muscle, and endothelial cells, and provided further evidence of the roles that CXCR7 may play within these cell types and the organs involved.

Collectively whilst our data demonstrate that administration of a selective monoclonal antibody directed towards CXCR7 inhibits cardiorenal fibrosis in the setting of mineraolocorticoid excess, the present study does not fully identify the mechanism in detail. Elucidation of the mode of action of the mAb requires extensive further study including an analysis of cell specific CXCR7 distribution and signalling together with its influence on the local levels relevant ligands. CXCR7 function is complex, with significant variation across cell and tissue distributions as recently reviewed by Duval and colleagues^38^. CXCR7 was initially considered to principally be a scavenger receptor for high affinity ligands such as CXCR12, thereby regulating extracellular levels of CXCL12. We have previously shown that the CXCR4 involved in cardiorenal fibrosis^25^. We did not investigate the effects of CXCR7 mAb on CXCL12 levels or the ability of the CXCR7 receptor in the presence of the mAb to bind CXCL12. Interestingly the expression of CXCR7 appeared to increase following antibody administration however more detailed studies of the cellular distribution and regulatory mechanism are needed.

Subsequent to the identification of CXCR7, it has also been identified that CXCL12 binding CXCR7 can lead to the activation of ERK and protein kinase B in a β-arrestin mediated manner^39^. Through such a mechanism, CXCR7 could potentially cause cardiac and renal fibrosis through activation of MAPK signalling pathways, however this requires further investigation. As such, antagonism of CXCR7 might represent a novel therapeutic strategy to improve heart and kidney function by attenuating fibrosis. In addition to CXCL12, several other endogenous ligands for CXCR7 have also been described, as reviewed^38,40^. Macrophage inhibitory factor (MIF) is a pro-inflammatory cytokine which has also been shown to be a key mediator of cardiac fibrosis, particularly in the context of myocardial ischemia. Interestingly, recent data indicate that MIF can mediate ERK activation in a CXCR7 mediated manner^41^. Adrenomedullin is a hormone known to be elevated in heart failure and has been found to bind to CXCR7, however it has not be shown to activate CXCR7 signalling. Other CXCR7 ligands include CXCL11 (also termed interferon-inducible T cell α-chemoattractant) and bovine adrenal medulla 22 peptide (BAM22) are also described but are likely not implicated in cardiorenal pathology given their tissue distribution^40^. In addition to direct effects on cardiac and renal cellular elements, the actions of the CXCR7 mAb may also have been mediated via effects on elements of the inflammatory pathway which we demonstrated to be activated in the DOCA-UNX model. As an example, the CXCL12/CXCR4 is activated in myocardial infarction and plays a key role in recruitment of inflammatory cells^42^, which may then exert profibrotic actions via release of cytokines including Il1 and Il6 .

The translational potential of our study has some additional limitations. Heart failure and chronic kidney disease are conditions associated with older age, and HFpEF has a greater prevalence in women. Accordingly future studies should include female and aged mice to investigate the relevance of the CXCR7 pathway in these situations. Furthermore, our study demonstrated the preventive action of CXCR7 antagonism, further studies will be required to investigate whether CXCR7 antagonism can reverse established cardiorenal fibrosis. Whilst our study demonstrates an anti-fibrotic and anti-inflammatory action of CXCR7 antagonism, we cannot resolve whether these findings are independent or linked.

## Conclusion

This study demonstrated that the CXCR7 axis pathway plays a significant role in cardiac and renal fibrosis in the setting of DOCA-UNX treatment. Antagonism of CXCR7 with a targetted monoclonal antibody can effectively ameliorate both cardiac and renal fibrosis and may represent an anti-fibrotic therapy in the setting of mineralocorticoid excess. Further study is needed to validate CXCR7 as a new anti-fibrotic therapy.

## Electronic supplementary material

Below is the link to the electronic supplementary material.

Supplementary Material 1

Supplementary Material 2

Supplementary Material 3

Supplementary Material 4

Supplementary Material 5

Supplementary Material 6

## Data Availability

Data generated during this study are available from the corresponding author on reasonable request.
