# Supplementary figures and images for: Chemokine receptor CXCR7 antagonism ameliorates cardiac and renal fibrosis induced by mineralocorticoid excess
Source: Sci Rep. 2024 Nov 6;14:26985. doi: 10.1038/s41598-024-75789-0 (PMC11541864; doi:10.1038/s41598-024-75789-0)

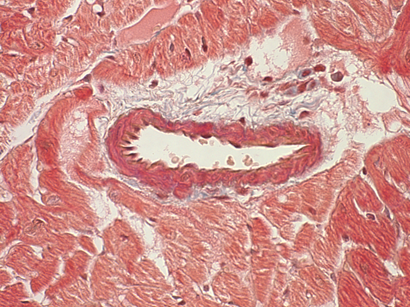

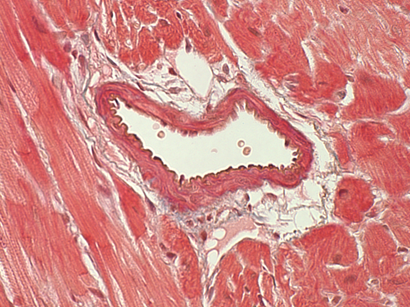

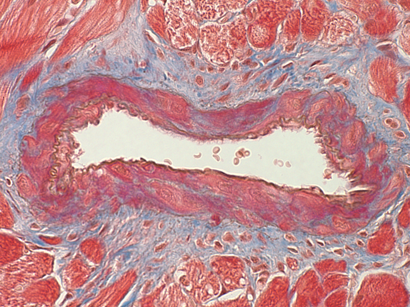

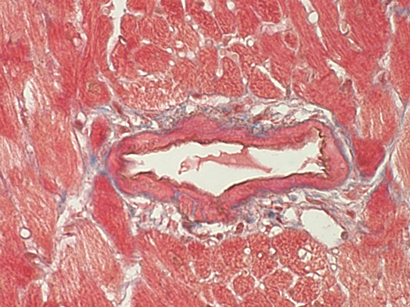


Control

Control+CXCR7

DOCA

DOCA+CXCR7

Supplement: Supplementary file 1 — Supplementary Material 1 [file 41598_2024_75789_MOESM1_ESM.docx]

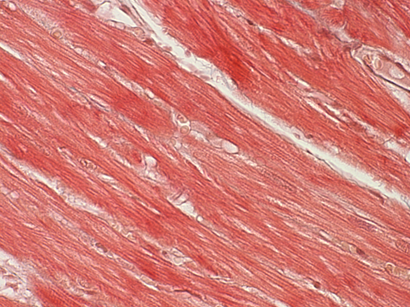

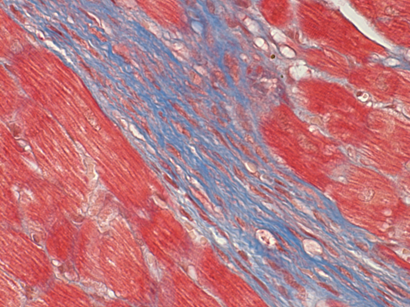

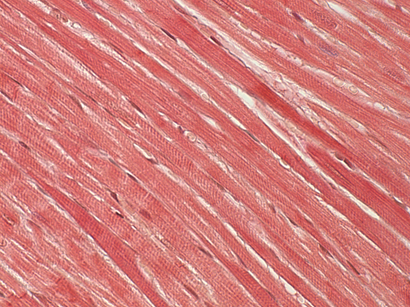

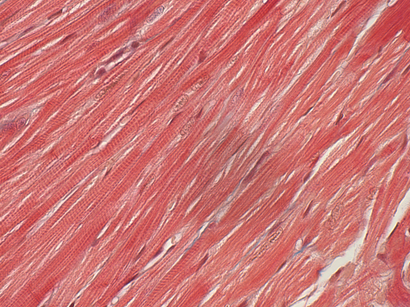


DOCA+CXCR7

DOCA

Control+CXCR7

Control

Supplement: Supplementary file 2 — Supplementary Material 2 [file 41598_2024_75789_MOESM2_ESM.docx]

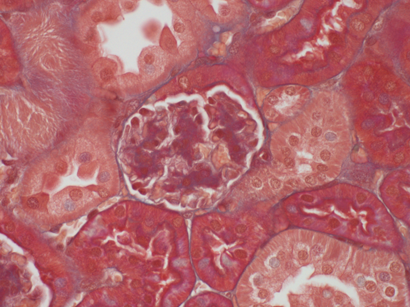

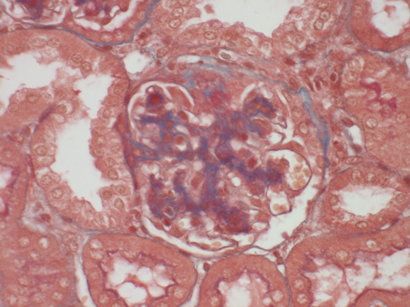

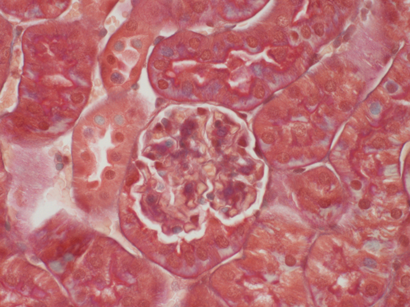

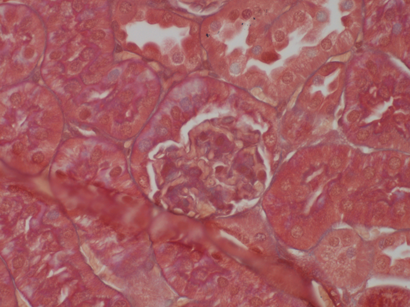


DOCA+CXCR7

DOCA

Control+CXCR7

Control

Supplement: Supplementary file 3 — Supplementary Material 3 [file 41598_2024_75789_MOESM3_ESM.docx]

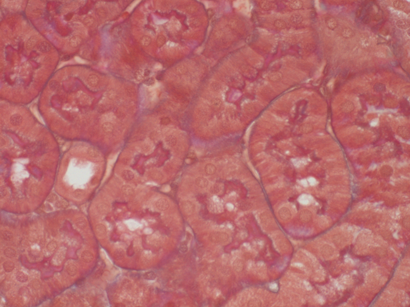

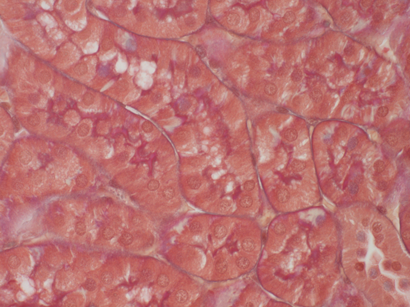

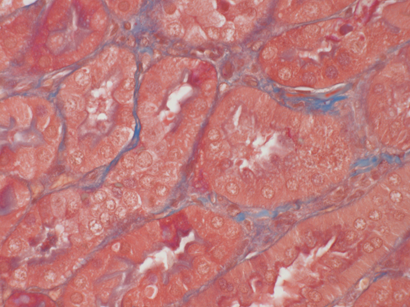

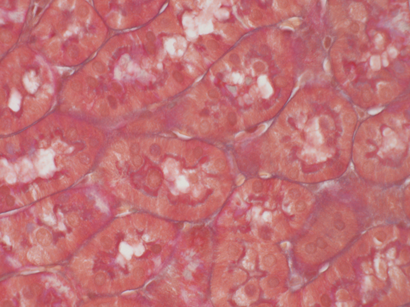


Control

Control+CXCR7

DOCA

DOCA+CXCR7

Supplement: Supplementary file 4 — Supplementary Material 4 [file 41598_2024_75789_MOESM4_ESM.docx]

Supplementary Figure 5


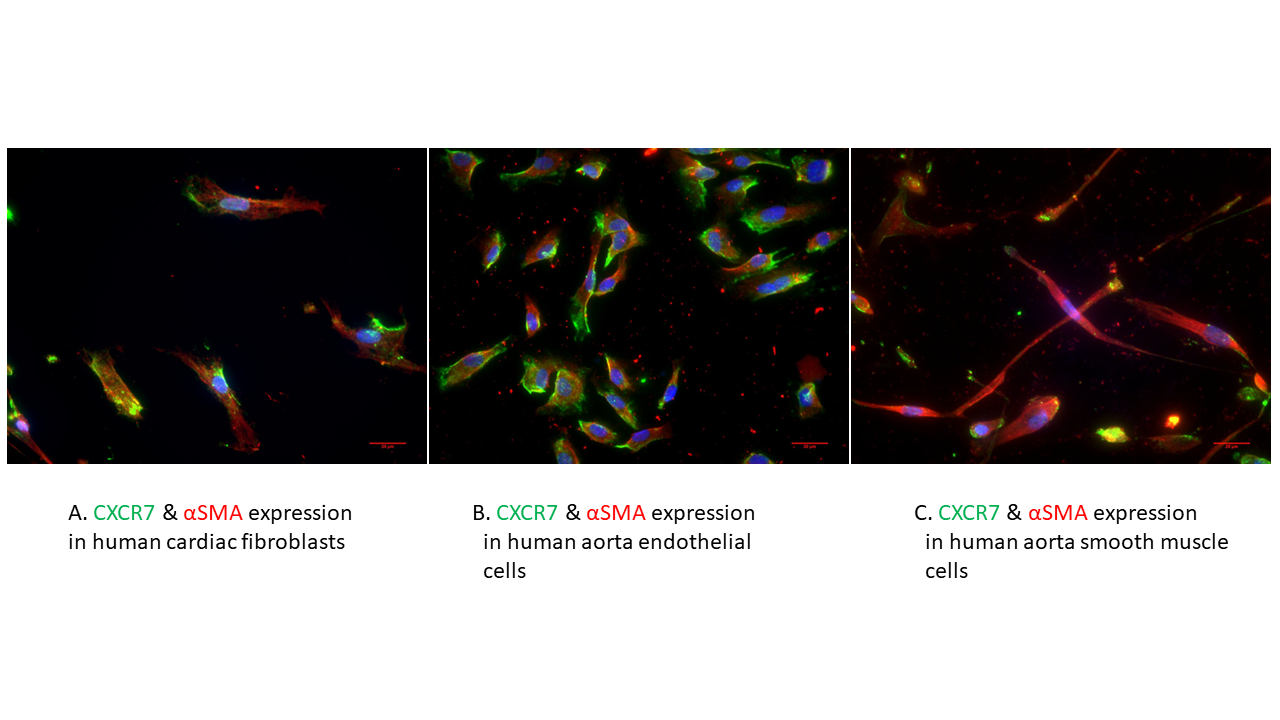

Supplement: Supplementary file 5 — Supplementary Material 5 [file 41598_2024_75789_MOESM5_ESM.docx]
