# Supplementary material for: Chemokine receptor CXCR7 antagonism ameliorates cardiac and renal fibrosis induced by mineralocorticoid excess
Source: Sci Rep. 2024 Nov 6;14:26985. doi: 10.1038/s41598-024-75789-0 (PMC11541864; doi:10.1038/s41598-024-75789-0)
Supplement: Supplementary file 6 — Supplementary Material 6 [file 41598_2024_75789_MOESM6_ESM.docx]

**Supplementary Figure Legends**

Figure 1. Representative photomicrographs of Masson’s-stained myocardial sections from Control, Control+CXCR7 mAb, DOCA and DOCA+CXCR7 mAb demonstrating perivascular fibrosis.

Figure 2. Representative photomicrographs of Masson’s-stained myocardial sections from Control, Control+CXCR7 mAb, DOCA and DOCA+CXCR7 mAb demonstrating interstitial fibrosis.

Figure 3. Representative photomicrographs of Masson’s-stained kidney sections from Control, Control+CXCR7 mAb, DOCA and DOCA+CXCR7 mAb demonstrating glomerular fibrosis.

Figure 4. Representative photomicrographs of Masson’s-stained kidney sections from Control, Control+CXCR7 mAb, DOCA and DOCA+CXCR7 mAb demonstrating tubulointerstitial fibrosis.

Figure 5. Immunohistochemical images showing CXCR7 and aSMA expression in cultured human cardiac fibroblasts, endothelial cells and smooth muscle cells.
